# Crystal structure of (2*S*,4*R*)-ethyl 4-nitro­methyl-1-[(*S*)-1-phenyl­eth­yl]-6-sulfanyl­idene­piperidine-2-carboxyl­ate

**DOI:** 10.1107/S2056989014026711

**Published:** 2015-01-01

**Authors:** Araceli Zárate, David Aparicio, Angel Palillero, Angel Mendoza

**Affiliations:** a Centro de Química, ICUAP, Benemérita Universidad Autónoma de Puebla, 72570, Puebla, Puebla, Mexico

**Keywords:** crystal structure, thio­piperidine, piperidine-2-thio­nes, hydrogen bonding

## Abstract

In the title compound, C_17_H_22_N_2_O_4_S, a thio­piperidine derivative, the piperidine ring has an envelope conformation with the methyl­ene C atom opposite to the C=S bond as the flap. The nitro­methyl substituent is equatorial while the eth­oxy­carbonyl group is axial. The mean planes of the nitro­methyl group, the carb­oxy group and phenyl ring are inclined to the mean plane through the five planar atoms of the piperidine ring [maximum deviation = 0.070 (4) Å] by 56.8 (2), 83.8 (5) and 87.1 (2)°, respectively. There is an intra­molecular C—H⋯O hydrogen bond involving an H atom of the eth­oxy­carbonyl group and a nitro O atom. In the crystal, mol­ecules are linked by C—H⋯O hydrogen bonds, forming chains along [100]. The chains are linked by further C—H⋯O hydrogen bonds, forming corrugated layers lying parallel to (001).

## Related literature   

For general background on piperidines and their derivatives, see: Poupart *et al.* (1999 ▸); Pinnick *et al.* (1990 ▸); Mukaiyama & Hoshino (1960 ▸); Ballini *et al.* (2007 ▸); Sośnicki (2009 ▸). For their biological activity, see: Leung *et al.* (2000 ▸). For their use in organometallic reactions, see: Tamaru *et al.* (1978 ▸, 1979 ▸). For details of the Cambridge Structural Database, see: Groom & Allen (2014 ▸).
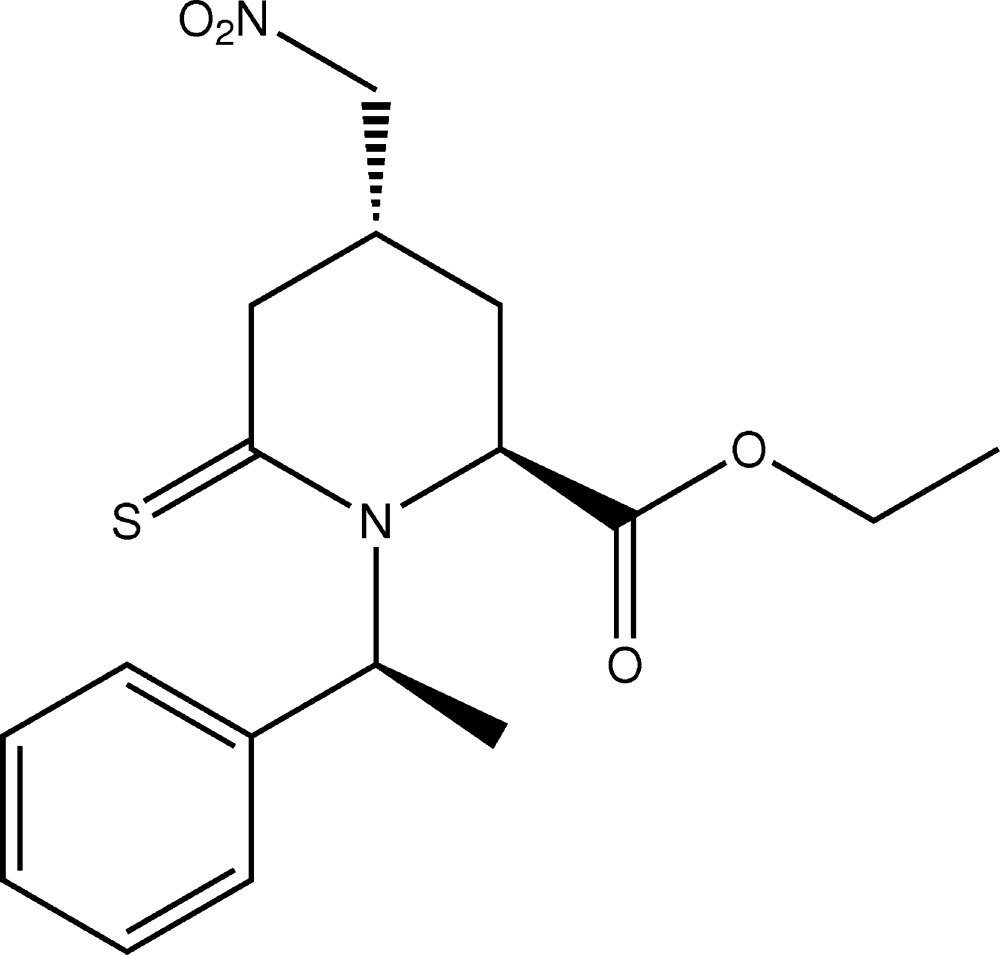


## Experimental   

### Crystal data  

C_17_H_22_N_2_O_4_S
*M* *_r_* = 350.42
Orthorhombic, 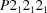

*a* = 5.7999 (2) Å
*b* = 10.0103 (6) Å
*c* = 30.4050 (18) Å
*V* = 1765.28 (16) Å^3^
*Z* = 4
Mo *K*α radiation
μ = 0.21 mm^−1^
*T* = 293 K
0.20 × 0.09 × 0.05 mm

### Data collection  

Agilent Xcalibur Atlas Gemini diffractometer
Absorption correction: analytical (*CrysAlis PRO*; Agilent, 2014 ▸) *T*_min_ = 0.979, *T*_max_ = 0.991
8486 measured reflections
3373 independent reflections
2306 reflections with *I* > 2σ(*I*)
*R*_int_ = 0.054

### Refinement  

*R*[*F* ^2^ > 2σ(*F* ^2^)] = 0.056
*wR*(*F* ^2^) = 0.104
*S* = 1.04
3373 reflections
219 parameters
H-atom parameters constrained
Δρ_max_ = 0.18 e Å^−3^
Δρ_min_ = −0.18 e Å^−3^
Absolute structure: Flack *x* determined using 705 quotients [(*I* ^+^)−(*I* ^−^)]/[(*I* ^+^)+(*I* ^−^)] (Parsons *et al.*, 2013 ▸)
Absolute structure parameter: 0.16 (8)

### 

Data collection: *CrysAlis PRO* (Agilent, 2014 ▸); cell refinement: *CrysAlis PRO*; data reduction: *CrysAlis PRO*; program(s) used to solve structure: *SHELXS2014* (Sheldrick, 2008 ▸); program(s) used to refine structure: *SHELXL2014* (Sheldrick, 2008 ▸); molecular graphics: *ORTEP-3 for Windows* (Farrugia, 2012 ▸) and *Mercury* (Macrae *et al.*, 2008 ▸); software used to prepare material for publication: *WinGX* (Farrugia, 2012 ▸), *PLATON* (Spek, 2009 ▸) and *publCIF* (Westrip, 2010 ▸).

## Supplementary Material

Crystal structure: contains datablock(s) global, I. DOI: 10.1107/S2056989014026711/su5036sup1.cif

Structure factors: contains datablock(s) I. DOI: 10.1107/S2056989014026711/su5036Isup2.hkl

**Fig. 1.**
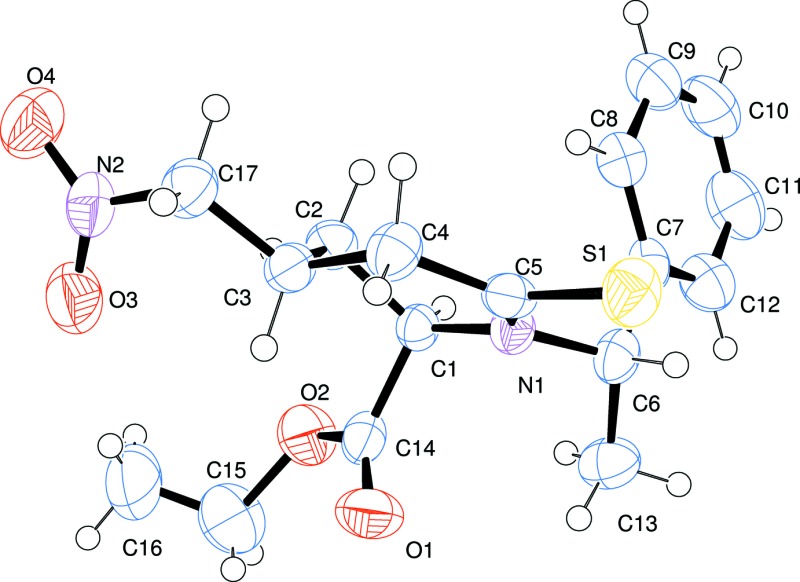
A view of the molecular structure of the title compound, with atom labelling. Displacement ellipsoids are drawn at the 30% probability level.

**Fig. 2.**
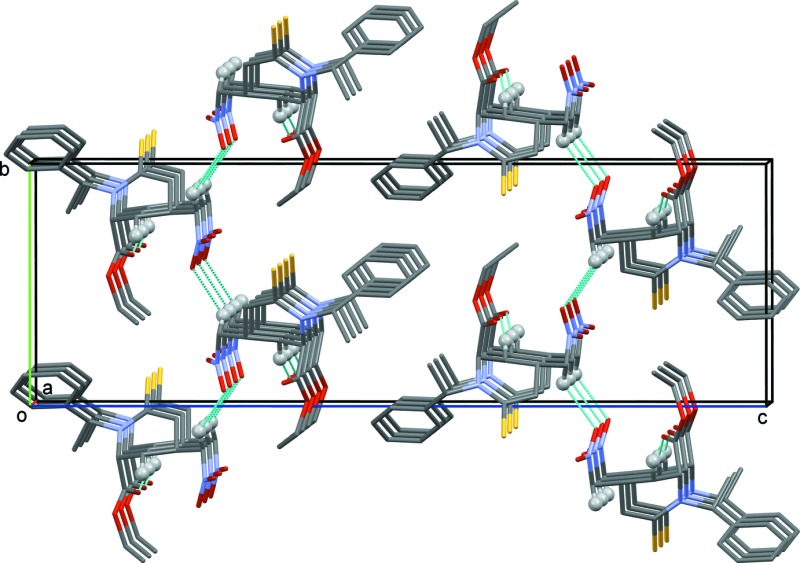
A view along the a axis of the crystal packing of the title compound. Hydrogen bonds are shown as dashed lines (see Table 1 for details; H atoms not involved in the intermolecular hydrogen bonding have been omitted for clarity).

. DOI: 10.1107/S2056989014026711/su5036fig1.tif
A view of the mol­ecular structure of the title compound, with atom labelling. Displacement ellipsoids are drawn at the 30% probability level.

a . DOI: 10.1107/S2056989014026711/su5036fig2.tif
A view along the *a* axis of the crystal packing of the title compound. Hydrogen bonds are shown as dashed lines (see Table 1 for details; H atoms not involved in the inter­molecular hydrogen bonding have been omitted for clarity).

CCDC reference: 1037775

Additional supporting information:  crystallographic information; 3D view; checkCIF report

## Figures and Tables

**Table 1:** Hydrogen-bond geometry (, )

*D*H*A*	*D*H	H*A*	*D* *A*	*D*H*A*
C16H16*A*O3	0.96	2.49	3.419(8)	163
C2H2*A*O1^i^	0.97	2.55	3.404(5)	147
C17H17*B*O3^ii^	0.97	2.58	3.380(6)	140

Symmetry codes: (i) 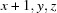; (ii) 
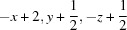
.

## supplementary crystallographic information

### S1. Comment

The Michael addition is one of the most important synthetic strategies performed in organic synthesis. Among the many applications, conjugate addition to α,β-unsaturated δ-lactams has been used in the synthesis of functionalized piperidines, due to a wide range of biological activities (Leung *et al.*, 2000). Similar to α,β-unsaturated δ-lactams, α,β-unsaturated δ-thiolactams are promising Michael acceptors affording 4-substituted piperidine-2-thiones (Sośnicki, 2009). They also form C—C bonds in the reaction with organometallics such as alkyllithium, alkylmagnesium (Tamaru *et al.*, 1978) and lithium enolates (Tamaru *et al.*, 1979). Among a broad range of nucleophiles applied to the C—C bond formation, the addition of aliphatic nitrocompounds play a significant role (Ballini *et al.*, 2007). In the presence of a base catalyst, the introduction of a nitroalkyl group into a α,β-unsaturated compound represent a key step in the preparation of chiral molecules due to versatile reactivity of the nitro functionality. The corresponding nitro compounds can be transformed into a wide range of synthetically valuable products such as amines (Poupart *et al.*, 1999), ketones (Pinnick *et al.*, 1990), carboxylic acids, nitrile oxides and other functionalities (Mukaiyama *et al.*, 1960).

In the title compound, Fig. 1, the piperidine ring has an envelope conformation with puckering parameters *Q* = 0.528 (4) Å, θ = 129.0 (4)°, φ = 314.6 (6)°, q_2_ = 0.411 (4)° and q_3_ = -0.332 (4)°. The phenyl-ethyl group linked atom N1 of the piperidine ring, shows a dihedral angle of 101.6 (4)° from the mean plane of the piperidine ring. The carboxyethyl group is placed in a axial position (torsion angle = 15.8 (3)°) and the nitromethyl group in an equatorial position (torsion angle = 73.7 (3)°) on the piperidine ring. The C5═S1 distance is 1.682 (4) Å, similar to that found for other piperidine-2-thiones (CSD; Groom & Allen, 2014). There is an intramolecular C-H···O hydrogen bond present (Table 1).

In the crystal, molecules are linked by C-H···O hydrogen bonds forming chains along [100], which are linked by further C-H···O hydrogen bonds forming corrugated layers lying parallel to (001); see Table 1 and Fig. 2.

### S2. Experimental

α,β-Unsaturated piperidine-2-thione derived from (*S*)-(-)-phenylethylamine (1.0 mmol) was dissolved in a solution of nitroalkane, and a catalytic amount of DBU was added. The mixture was stirred at room temperature for 2 h. When the reaction was complete, 5 ml of concentrated NH_4_Cl was added and the solution was extracted twice with ethyl acetate. The organic phase was dried, filtered, and concentrated *in vacuo*. The crude product was purified by column chromatography on silica (petroleum ether/ethyl acetate 80:20) giving the title compound as a white solid (yield 80%; m.p. 383- 385 K). It was crystallized using petroleum ether/dichloromethane, giving colourless prismatic crystals. [α]D^20^= -92.3 (c 1.0, CH_2_Cl_2_). IR (KBr pellet, cm^-1^): ν= 3746, 2977, 2929, 1739, 1551, 1461, 1196, 1075, 701, 548. ^1^H NMR (500 MHz, CDCl_3_): δ 1.31 (t, *J* = 7.1 Hz, 3H), 1.33 (m, 1H), 1.52 (d, *J* = 7.1 Hz, 3H), 2.25 (ddd, *J* = 1.9, 5.7, 13.7 Hz, 1H), 2.79 (m, 1H), 2.99 (dd, *J* = 7.2, 18.3 Hz, 1H), 3.37 (ddd, *J* = 0.6, 7.7, 18.3 Hz, 1H), 4.05 (dd, *J* = 2.4, 5.4 Hz, 1H), 4.21 (dd, *J* = 8.4, 12.8 Hz, 1H), 4.27 (m, 2H), 4.35 (dd, *J* = 6.1, 12.8 Hz, 1H), 7.35 (m, 5H). ^13^C NMR (100 MHz, CDCl_3_) δ 14.1, 14.5, 28.5, 29.9, 43.4, 55.6, 58.4, 62.5, 78.8, 127.0–129.0, 138.2, 170.0, 199.1.

### S3. Refinement

Crystal data, data collection and structure refinement details are summarized in Table 2. The C-bound H atoms were placed in idealized positions and refined as riding on their parent atoms, with C–H = 0.93–0.98 Å and with *U*_iso_(H) = 1.5*U*_eq_(C) for methyl H atoms and = 1.2*U*_eq_(C) for other H atoms. In the final cycles of refinement 18 reflections were omitted owing to poor agreement.

### Crystal data

**Table:**

C_17_H_22_N_2_O_4_S	*F*(000) = 744
*M**_r_* = 350.42	*D*_x_ = 1.319 Mg m^−^^3^
Orthorhombic, *P*2_1_2_1_2_1_	Mo *K*α radiation, λ = 0.71073 Å
Hall symbol: P 2ac 2ab	Cell parameters from 2144 reflections
*a* = 5.7999 (2) Å	θ = 3.6–22.4°
*b* = 10.0103 (6) Å	µ = 0.21 mm^−^^1^
*c* = 30.4050 (18) Å	*T* = 293 K
*V* = 1765.28 (16) Å^3^	Prism, colourless
*Z* = 4	0.20 × 0.09 × 0.05 mm

### Data collection

**Table:**

Agilent Xcalibur Atlas Gemini diffractometer	3373 independent reflections
Radiation source: Enhance (Mo) X-ray Source	2306 reflections with *I* > 2σ(*I*)
Graphite monochromator	*R*_int_ = 0.054
Detector resolution: 10.5564 pixels mm^-1^	θ_max_ = 26.1°, θ_min_ = 2.9°
w scans	*h* = −7→6
Absorption correction: analytical (*CrysAlis PRO*; Agilent, 2014)	*k* = −12→12
*T*_min_ = 0.979, *T*_max_ = 0.991	*l* = −37→28
8486 measured reflections	

### Refinement

**Table:**

Refinement on *F*^2^	Hydrogen site location: inferred from neighbouring sites
Least-squares matrix: full	H-atom parameters constrained
*R*[*F*^2^ > 2σ(*F*^2^)] = 0.056	*w* = 1/[σ^2^(*F*_o_^2^) + (0.0336*P*)^2^ + 0.2223*P*] where *P* = (*F*_o_^2^ + 2*F*_c_^2^)/3
*wR*(*F*^2^) = 0.104	(Δ/σ)_max_ < 0.001
*S* = 1.04	Δρ_max_ = 0.18 e Å^−^^3^
3373 reflections	Δρ_min_ = −0.18 e Å^−^^3^
219 parameters	Absolute structure: Flack *x* determined using 705 quotients [(*I*^+^)-(*I*^-^)]/[(*I*^+^)+(*I*^-^)] (Parsons *et al.*, 2013)
0 restraints	Absolute structure parameter: 0.16 (8)

### Special details

**Table:**

Geometry. All e.s.d.'s (except the e.s.d. in the dihedral angle between two l.s. planes) are estimated using the full covariance matrix. The cell e.s.d.'s are taken into account individually in the estimation of e.s.d.'s in distances, angles and torsion angles; correlations between e.s.d.'s in cell parameters are only used when they are defined by crystal symmetry. An approximate (isotropic) treatment of cell e.s.d.'s is used for estimating e.s.d.'s involving l.s. planes.

### Fractional atomic coordinates and isotropic or equivalent isotropic displacement parameters (Å^2^)

**Table:**

	*x*	*y*	*z*	*U*_iso_*/*U*_eq_	
S1	0.19570 (19)	0.59698 (11)	0.34603 (4)	0.0521 (3)	
O1	0.2788 (5)	0.1293 (3)	0.35882 (11)	0.0613 (9)	
C1	0.5823 (6)	0.2794 (4)	0.38339 (13)	0.0335 (9)	
H1	0.6554	0.2775	0.4124	0.04*	
N1	0.4157 (5)	0.3912 (3)	0.38245 (10)	0.0348 (8)	
O3	0.8838 (6)	0.0880 (4)	0.27210 (11)	0.0706 (10)	
C2	0.7699 (6)	0.2959 (4)	0.34888 (12)	0.0401 (10)	
H2A	0.8704	0.2185	0.3489	0.048*	
H2B	0.8622	0.3743	0.3554	0.048*	
C6	0.3122 (7)	0.4254 (4)	0.42575 (13)	0.0424 (10)	
H6	0.1929	0.4925	0.42	0.051*	
C3	0.6547 (7)	0.3112 (4)	0.30381 (13)	0.0387 (10)	
H3	0.5528	0.2346	0.2988	0.046*	
O2	0.6039 (5)	0.0483 (3)	0.38838 (11)	0.0661 (10)	
N2	0.9716 (7)	0.1947 (4)	0.26262 (12)	0.0515 (10)	
C7	0.4909 (7)	0.4921 (4)	0.45523 (15)	0.0411 (10)	
C5	0.3778 (6)	0.4662 (4)	0.34676 (14)	0.0379 (10)	
C13	0.1904 (8)	0.3059 (5)	0.44649 (14)	0.0544 (12)	
H13A	0.0961	0.2627	0.4248	0.082*	
H13B	0.0952	0.3359	0.4704	0.082*	
H13C	0.3031	0.244	0.4574	0.082*	
C14	0.4629 (7)	0.1445 (4)	0.37579 (14)	0.0410 (10)	
C17	0.8290 (7)	0.3186 (4)	0.26649 (14)	0.0508 (11)	
H17A	0.7477	0.3333	0.239	0.061*	
H17B	0.93	0.3944	0.2713	0.061*	
O4	1.1672 (6)	0.2065 (4)	0.24925 (12)	0.0746 (10)	
C4	0.5101 (7)	0.4380 (4)	0.30508 (14)	0.0441 (11)	
H4A	0.6114	0.5132	0.2995	0.053*	
H4B	0.4006	0.4341	0.281	0.053*	
C12	0.4732 (8)	0.4867 (5)	0.50057 (16)	0.0576 (13)	
H12	0.3535	0.4388	0.5134	0.069*	
C8	0.6741 (8)	0.5636 (4)	0.43755 (15)	0.0524 (12)	
H8	0.6916	0.5674	0.4072	0.063*	
C11	0.6311 (10)	0.5515 (5)	0.52687 (17)	0.0707 (15)	
H11	0.616	0.5467	0.5573	0.085*	
C9	0.8308 (8)	0.6293 (5)	0.46405 (19)	0.0646 (14)	
H9	0.9505	0.6778	0.4514	0.078*	
C10	0.8102 (10)	0.6231 (5)	0.50910 (19)	0.0708 (15)	
H10	0.9156	0.6666	0.5272	0.085*	
C15	0.5480 (11)	−0.0877 (5)	0.3739 (3)	0.107 (2)	
H15A	0.5214	−0.1435	0.3995	0.128*	
H15B	0.4069	−0.0858	0.3568	0.128*	
C16	0.7207 (13)	−0.1424 (7)	0.3491 (2)	0.138 (3)	
H16A	0.7451	−0.0886	0.3233	0.207*	
H16B	0.678	−0.2313	0.3404	0.207*	
H16C	0.8602	−0.1458	0.366	0.207*	

### Atomic displacement parameters (Å^2^)

**Table:**

	*U*^11^	*U*^22^	*U*^33^	*U*^12^	*U*^13^	*U*^23^
S1	0.0540 (6)	0.0423 (6)	0.0600 (8)	0.0143 (6)	0.0054 (6)	0.0103 (6)
O1	0.0488 (17)	0.053 (2)	0.082 (2)	−0.0121 (15)	−0.0068 (17)	−0.0028 (16)
C1	0.031 (2)	0.034 (2)	0.036 (2)	0.0039 (18)	−0.0003 (19)	−0.0017 (18)
N1	0.0360 (16)	0.0332 (19)	0.035 (2)	0.0034 (15)	0.0024 (15)	0.0013 (16)
O3	0.096 (3)	0.049 (2)	0.067 (2)	0.003 (2)	0.0215 (19)	−0.0059 (18)
C2	0.034 (2)	0.042 (2)	0.045 (2)	0.0007 (18)	−0.005 (2)	−0.002 (2)
C6	0.039 (2)	0.042 (2)	0.047 (3)	0.010 (2)	0.004 (2)	0.0018 (19)
C3	0.036 (2)	0.041 (2)	0.039 (2)	−0.0018 (19)	0.002 (2)	0.0004 (19)
O2	0.0655 (19)	0.0378 (18)	0.095 (3)	0.0108 (17)	0.004 (2)	0.0027 (17)
N2	0.059 (2)	0.056 (3)	0.039 (2)	0.006 (2)	0.008 (2)	−0.0078 (19)
C7	0.048 (2)	0.032 (2)	0.043 (3)	0.008 (2)	0.003 (2)	−0.005 (2)
C5	0.0345 (19)	0.033 (2)	0.047 (3)	−0.0050 (17)	−0.003 (2)	0.001 (2)
C13	0.052 (2)	0.063 (3)	0.048 (3)	−0.011 (3)	0.004 (2)	0.005 (2)
C14	0.044 (2)	0.038 (2)	0.041 (3)	0.005 (2)	0.012 (2)	0.003 (2)
C17	0.056 (2)	0.047 (3)	0.049 (3)	0.001 (2)	0.012 (2)	0.003 (2)
O4	0.0502 (18)	0.092 (3)	0.082 (2)	0.0043 (19)	0.014 (2)	−0.014 (2)
C4	0.045 (2)	0.045 (3)	0.043 (3)	0.004 (2)	0.005 (2)	0.006 (2)
C12	0.068 (3)	0.052 (3)	0.053 (3)	0.000 (3)	−0.001 (3)	−0.003 (2)
C8	0.054 (2)	0.048 (3)	0.056 (3)	−0.001 (2)	0.006 (3)	−0.011 (2)
C11	0.098 (4)	0.063 (3)	0.051 (3)	0.007 (3)	−0.014 (3)	−0.011 (3)
C9	0.063 (3)	0.052 (3)	0.079 (4)	−0.006 (3)	−0.001 (3)	−0.018 (3)
C10	0.077 (3)	0.057 (3)	0.078 (4)	−0.003 (3)	−0.019 (3)	−0.021 (3)
C15	0.105 (4)	0.032 (3)	0.184 (7)	0.008 (3)	0.034 (5)	−0.014 (4)
C16	0.166 (7)	0.073 (4)	0.176 (7)	−0.028 (5)	0.101 (6)	−0.048 (4)

### Geometric parameters (Å, º)

**Table:**

S1—C5	1.682 (4)	C5—C4	1.508 (5)
O1—C14	1.195 (5)	C13—H13A	0.96
C1—H1	0.98	C13—H13B	0.96
C1—N1	1.479 (5)	C13—H13C	0.96
C1—C2	1.521 (5)	C17—H17A	0.97
C1—C14	1.535 (5)	C17—H17B	0.97
N1—C6	1.487 (5)	C4—H4A	0.97
N1—C5	1.338 (5)	C4—H4B	0.97
O3—N2	1.218 (5)	C12—H12	0.93
C2—H2A	0.97	C12—C11	1.378 (7)
C2—H2B	0.97	C8—H8	0.93
C2—C3	1.532 (5)	C8—C9	1.381 (6)
C6—H6	0.98	C11—H11	0.93
C6—C7	1.524 (6)	C11—C10	1.372 (7)
C6—C13	1.526 (6)	C9—H9	0.93
C3—H3	0.98	C9—C10	1.376 (7)
C3—C17	1.521 (5)	C10—H10	0.93
C3—C4	1.522 (5)	C15—H15A	0.97
O2—C14	1.321 (5)	C15—H15B	0.97
O2—C15	1.467 (6)	C15—C16	1.369 (8)
N2—C17	1.495 (5)	C16—H16A	0.96
N2—O4	1.211 (4)	C16—H16B	0.96
C7—C12	1.383 (6)	C16—H16C	0.96
C7—C8	1.390 (6)		
			
N1—C1—H1	108.3	O1—C14—C1	125.5 (4)
N1—C1—C2	111.8 (3)	O1—C14—O2	125.8 (4)
N1—C1—C14	111.6 (3)	O2—C14—C1	108.6 (3)
C2—C1—H1	108.3	C3—C17—H17A	109.1
C2—C1—C14	108.4 (3)	C3—C17—H17B	109.1
C14—C1—H1	108.3	N2—C17—C3	112.7 (3)
C1—N1—C6	114.9 (3)	N2—C17—H17A	109.1
C5—N1—C1	123.2 (3)	N2—C17—H17B	109.1
C5—N1—C6	121.5 (3)	H17A—C17—H17B	107.8
C1—C2—H2A	110	C3—C4—H4A	108
C1—C2—H2B	110	C3—C4—H4B	108
C1—C2—C3	108.4 (3)	C5—C4—C3	117.3 (3)
H2A—C2—H2B	108.4	C5—C4—H4A	108
C3—C2—H2A	110	C5—C4—H4B	108
C3—C2—H2B	110	H4A—C4—H4B	107.2
N1—C6—H6	106.5	C7—C12—H12	119.7
N1—C6—C7	110.3 (3)	C11—C12—C7	120.7 (5)
N1—C6—C13	111.8 (3)	C11—C12—H12	119.7
C7—C6—H6	106.5	C7—C8—H8	119.2
C7—C6—C13	114.5 (3)	C9—C8—C7	121.5 (4)
C13—C6—H6	106.5	C9—C8—H8	119.2
C2—C3—H3	108.9	C12—C11—H11	119.3
C17—C3—C2	112.5 (3)	C10—C11—C12	121.3 (5)
C17—C3—H3	108.9	C10—C11—H11	119.3
C17—C3—C4	110.2 (3)	C8—C9—H9	119.9
C4—C3—C2	107.5 (3)	C10—C9—C8	120.1 (5)
C4—C3—H3	108.9	C10—C9—H9	119.9
C14—O2—C15	117.0 (4)	C11—C10—C9	118.8 (5)
O3—N2—C17	118.5 (4)	C11—C10—H10	120.6
O4—N2—O3	123.8 (4)	C9—C10—H10	120.6
O4—N2—C17	117.6 (4)	O2—C15—H15A	109.2
C12—C7—C6	121.2 (4)	O2—C15—H15B	109.2
C12—C7—C8	117.5 (4)	H15A—C15—H15B	107.9
C8—C7—C6	121.2 (4)	C16—C15—O2	112.0 (5)
N1—C5—S1	123.4 (3)	C16—C15—H15A	109.2
N1—C5—C4	119.5 (3)	C16—C15—H15B	109.2
C4—C5—S1	117.0 (3)	C15—C16—H16A	109.5
C6—C13—H13A	109.5	C15—C16—H16B	109.5
C6—C13—H13B	109.5	C15—C16—H16C	109.5
C6—C13—H13C	109.5	H16A—C16—H16B	109.5
H13A—C13—H13B	109.5	H16A—C16—H16C	109.5
H13A—C13—H13C	109.5	H16B—C16—H16C	109.5
H13B—C13—H13C	109.5		
			
S1—C5—C4—C3	−173.4 (3)	C6—C7—C12—C11	−177.6 (4)
C1—N1—C6—C7	71.5 (4)	C6—C7—C8—C9	177.1 (4)
C1—N1—C6—C13	−57.2 (4)	C7—C12—C11—C10	−0.1 (7)
C1—N1—C5—S1	−177.7 (3)	C7—C8—C9—C10	1.1 (7)
C1—N1—C5—C4	−0.3 (5)	C5—N1—C6—C7	−101.6 (4)
C1—C2—C3—C17	−175.9 (3)	C5—N1—C6—C13	129.7 (4)
C1—C2—C3—C4	62.6 (4)	C13—C6—C7—C12	−27.2 (5)
N1—C1—C2—C3	−55.8 (4)	C13—C6—C7—C8	154.6 (4)
N1—C1—C14—O1	20.7 (6)	C14—C1—N1—C6	89.9 (4)
N1—C1—C14—O2	−163.5 (3)	C14—C1—N1—C5	−97.1 (4)
N1—C6—C7—C12	−154.4 (4)	C14—C1—C2—C3	67.6 (4)
N1—C6—C7—C8	27.4 (5)	C14—O2—C15—C16	120.1 (6)
N1—C5—C4—C3	9.0 (5)	C17—C3—C4—C5	−162.9 (3)
O3—N2—C17—C3	32.8 (5)	O4—N2—C17—C3	−148.7 (4)
C2—C1—N1—C6	−148.5 (3)	C4—C3—C17—N2	−178.1 (3)
C2—C1—N1—C5	24.4 (5)	C12—C7—C8—C9	−1.2 (6)
C2—C1—C14—O1	−102.9 (4)	C12—C11—C10—C9	−0.1 (8)
C2—C1—C14—O2	72.9 (4)	C8—C7—C12—C11	0.7 (7)
C2—C3—C17—N2	62.0 (5)	C8—C9—C10—C11	−0.4 (8)
C2—C3—C4—C5	−40.0 (4)	C15—O2—C14—O1	10.7 (7)
C6—N1—C5—S1	−5.2 (5)	C15—O2—C14—C1	−165.2 (4)
C6—N1—C5—C4	172.2 (3)		

### Hydrogen-bond geometry (Å, º)

**Table:**

*D*—H···*A*	*D*—H	H···*A*	*D*···*A*	*D*—H···*A*
C16—H16*A*···O3	0.96	2.49	3.419 (8)	163
C2—H2*A*···O1^i^	0.97	2.55	3.404 (5)	147
C17—H17*B*···O3^ii^	0.97	2.58	3.380 (6)	140

Symmetry codes: (i) *x*+1, *y*, *z*; (ii) −*x*+2, *y*+1/2, −*z*+1/2.
