# Supplementary figures and images for: Crystal structure of (2S,4R)-ethyl 4-nitro­methyl-1-[(S)-1-phenyl­eth­yl]-6-sulfanyl­idene­piperidine-2-carboxyl­ate
Source: Acta Crystallogr E Crystallogr Commun. 2015 Jan 1;71(Pt 1):o41–2. doi: 10.1107/S2056989014026711 (PMC4331902; doi:10.1107/S2056989014026711)

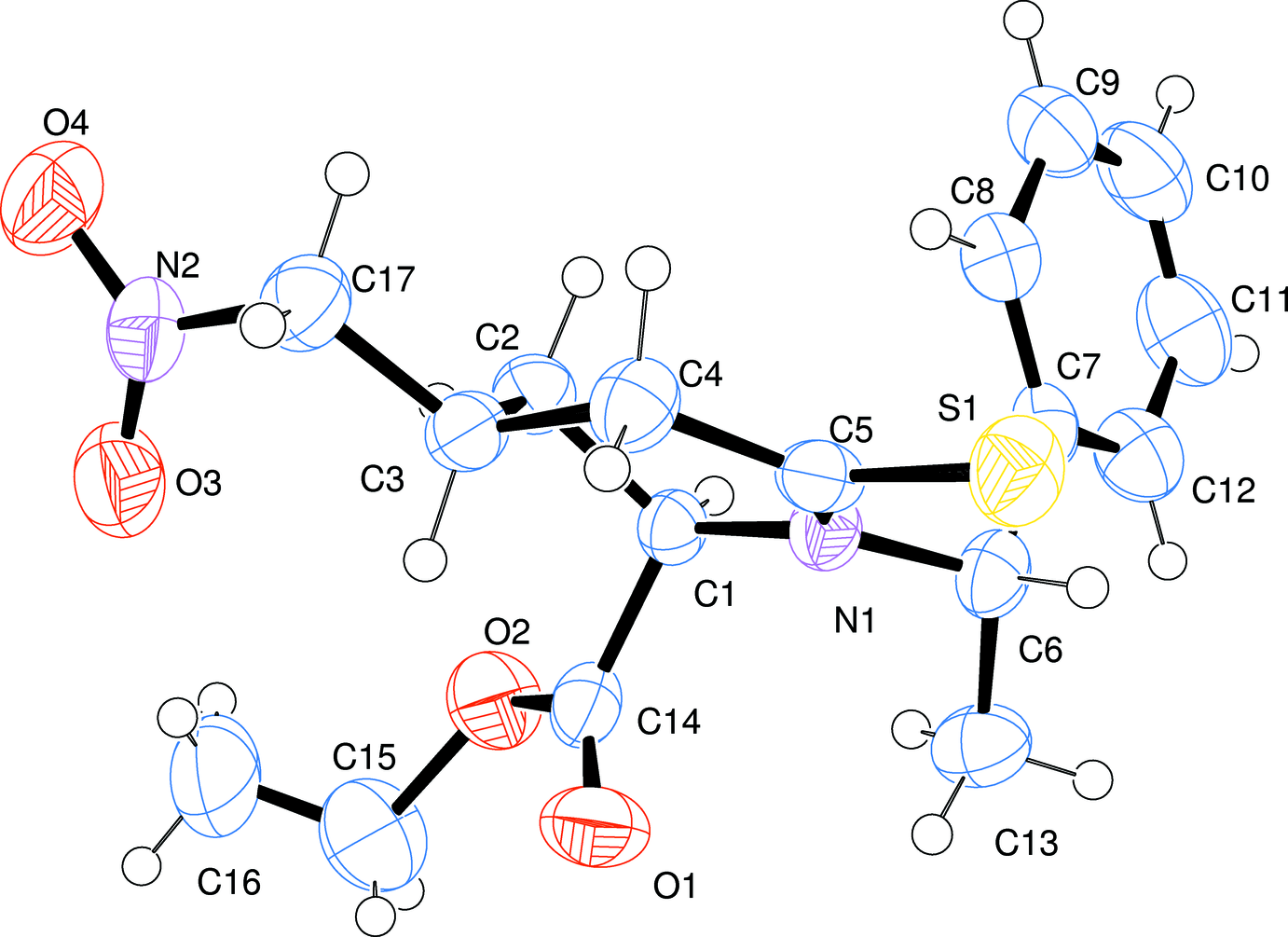

Supplement: Supplementary file 3 [file e-71-00o41-fig1.tif]

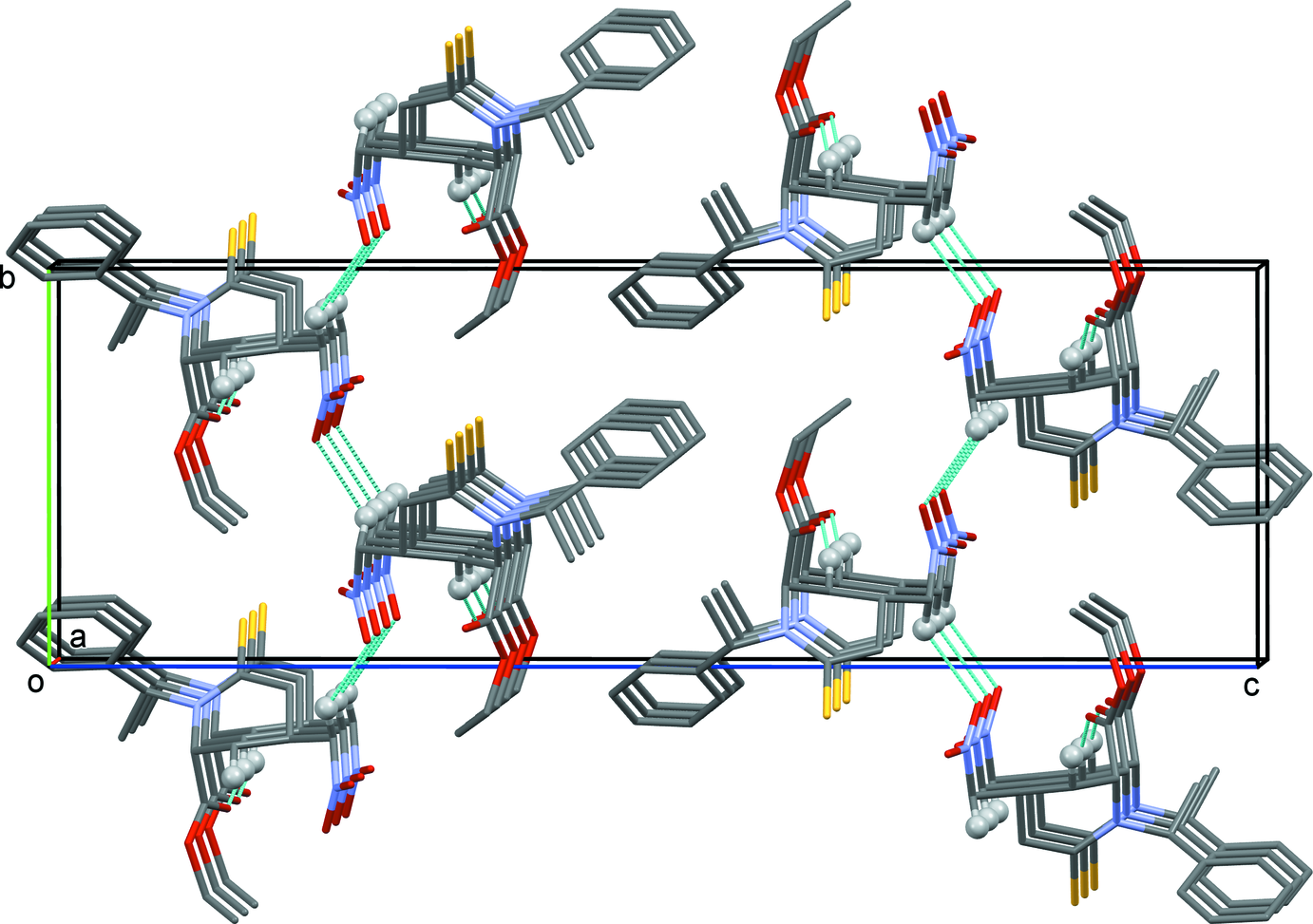

Supplement: Supplementary file 4 [file e-71-00o41-fig2.tif]
